# Risk factors for osteonecrosis of the femoral head in brain tumor patients receiving corticosteroid after surgery

**DOI:** 10.1371/journal.pone.0238368

**Published:** 2020-09-03

**Authors:** Seung-Jae Lim, Ingwon Yeo, Chan-Woo Park, Hyeon Lee, Youn-Soo Park, Jung-Il Lee

**Affiliations:** 1 Department of Orthopedic Surgery, Samsung Medical Center, Sungkyunkwan University School of Medicine, Seoul, Republic of Korea; 2 Department of Orthopedic Surgery, Sejong General Hospital, Bucheon, Republic of Korea; 3 Department of Neurosurgery, Samsung Medical Center, Sungkyunkwan University School of Medicine, Seoul, Republic of Korea; Stanford University, UNITED STATES

## Abstract

**Purpose:**

Non-traumatic osteonecrosis of the femoral head (ONFH) is a plausible complication in brain tumor patients. Frequent use of corticosteroid therapy, chemotherapy, and oxidative stress for managing brain tumors may be associated with the development of ONFH. However, there is little knowledge on the prevalence and risk factors of ONFH from brain tumor. This study aimed to investigate the prevalence and risk factors of ONFH in patients with primary brain tumors.

**Methods:**

This retrospective cohort study included data from consecutive patients between December 2005 and August 2016 from a tertiary university hospital in South Korea. A total of 73 cases of ONFH were identified among 10,674 primary brain tumor patients. After excluding subjects (25 out of 73) with missing data, history of alcohol consumption or smoking, history of femoral bone trauma or surgery, comorbidities such as systemic lupus erythematosus (SLE), sickle cell disease, cancer patients other than brain tumor, and previous diagnosis of contralateral ONFH, we performed a 1:2 propensity score-matched, case–control study (ONFH group, 48; control group, 96). Risk factors of ONFH in primary brain tumor were evaluated by univariate and multivariate logistic regression analyses.

**Results:**

The prevalence of ONFH in patients with surgical resection of primary brain tumor was 683.9 per 100,000 persons (73 of 10,674). In this cohort, 55 of 74 patients (74.3%) underwent THA for ONFH treatment. We found that diabetes was an independent factor associated with an increased risk of ONFH in primary brain tumor patients (OR = 7.201, 95% CI, 1.349–38.453, p = 0.021). There was a significant difference in univariate analysis, including panhypopituitarism (OR = 4.394, 95% CI, 1.794–11.008, p = 0.002), supratentorial location of brain tumor (OR = 2.616, 95% CI, 1.245–5.499, p = 0.011), and chemotherapy (OR = 2.867, 95% CI, 1.018–8.069, p = 0.046).

**Conclusions:**

This study demonstrated that the prevalence of ONFH after surgical resection of primary brain tumor was 0.68%. Diabetes was an independent risk factor for developing ONFH, whereas corticosteroid dose was not. Routine screening for brain tumor-associated ONFH is not recommended; however, a high index of clinical suspicion in these patients at risk may allow for early intervention and preservation of the joints.

## Introduction

Osteonecrosis of the femoral head (ONFH) is a debilitating complication associated with an interruption of the blood supply to a segment of the femoral head, which subsequently undergoes necrosis and collapse, irreversible destruction of the hip joint, and leading to total hip arthroplasty (THA) [1, 2]. Non-traumatic ONFH is thought to be a multifactorial disease. Although corticosteroid exposure and alcohol abuse are considered major risk factors, other factors such as sickle cell disease, systemic lupus erythematosus (SLE), Caisson disease, HIV and smoking are also associated [3, 4]. The pathogenesis of ONFH remains unclear, however, several reports have suggested possible pathogenesis, such as abnormality of lipid metabolism, thrombosis, and oxidative stress [5–7]. Several cancer treatment regimens also have been reported to be associated with the development of osteonecrosis, including radiation therapy; corticosteroid medications; immunotherapy, including anti-angiogenic agents; and several chemotherapeutic agents [8, 9].

Non-traumatic ONFH is recognized as a potential complication in solid-tumor cancer patients receiving treatment with or without corticosteroids therapy [9–14]. Corticosteroids, a known risk factor for ONFH, are frequently used primarily to suppress peritumor edema and the mass effect in brain tumor patients [15]. For this, few observational studies evaluated the risk of ONFH in brain tumor patients receiving perioperative corticosteroid [16]. Furthermore, pituitary dysfunction, a devastating sequelae of brain tumor, has been reported to be associated with ONFH in brain tumor patients [17, 18]. Recently, Lim et al. reported favorable clinical results and high patient satisfaction in total hip arthroplasty (THA) performed on corticosteroid-induced ONFH after surgical removal of a primary brain tumor [19].

As the long-term survival of patients with solid tumors increases, the prevalence of brain tumor-related ONFH may also increase. Patients should be informed that ONFH is a potential complication of brain tumor treatment. Measures to reduce risk should be taken, and patients should be monitored for early symptoms. It also appears that patients at higher risk for ONFH are identifiable. However, the clinical relationship between ONFH and brain tumor has not been fully characterized. In this study, we investigated (1) the prevalence of ONFH in primary brain tumor patients to describe patient characteristics related to the development of ONFH, and (2) identified risk factors of ONFH in these patients.

## Materials and methods

### Study design

This was a retrospective comparative, propensity score-matched study using the medical records of Samsung Medical Center, a tertiary referral hospital in Korea. The first objective of this study was to estimate the prevalence of ONFH in primary brain tumor patients. All consecutive patients who underwent treatment for a brain tumor between December 2005 and August 2016 (n = 13,171) were included initially. Before evaluating the prevalence of ONFH from these population, we excluded patients who were diagnosed with metastatic brain tumors, and lost to regular outpatient follow-up (n = 2,497), resulting in final 10,674 primary brain tumor patients population. Among this population, we identified the number of patients with newly developed ONFH during the outpatient follow-up period after brain tumor treatment. Demographic information (age, sex, BMI), comorbidities, and the data on diagnosis and treatment of brain tumor and ONFH were collected from the hospital information or medical record system. We described characteristics of primary brain tumor patients with newly developed ONFH.

**Fig 1:**
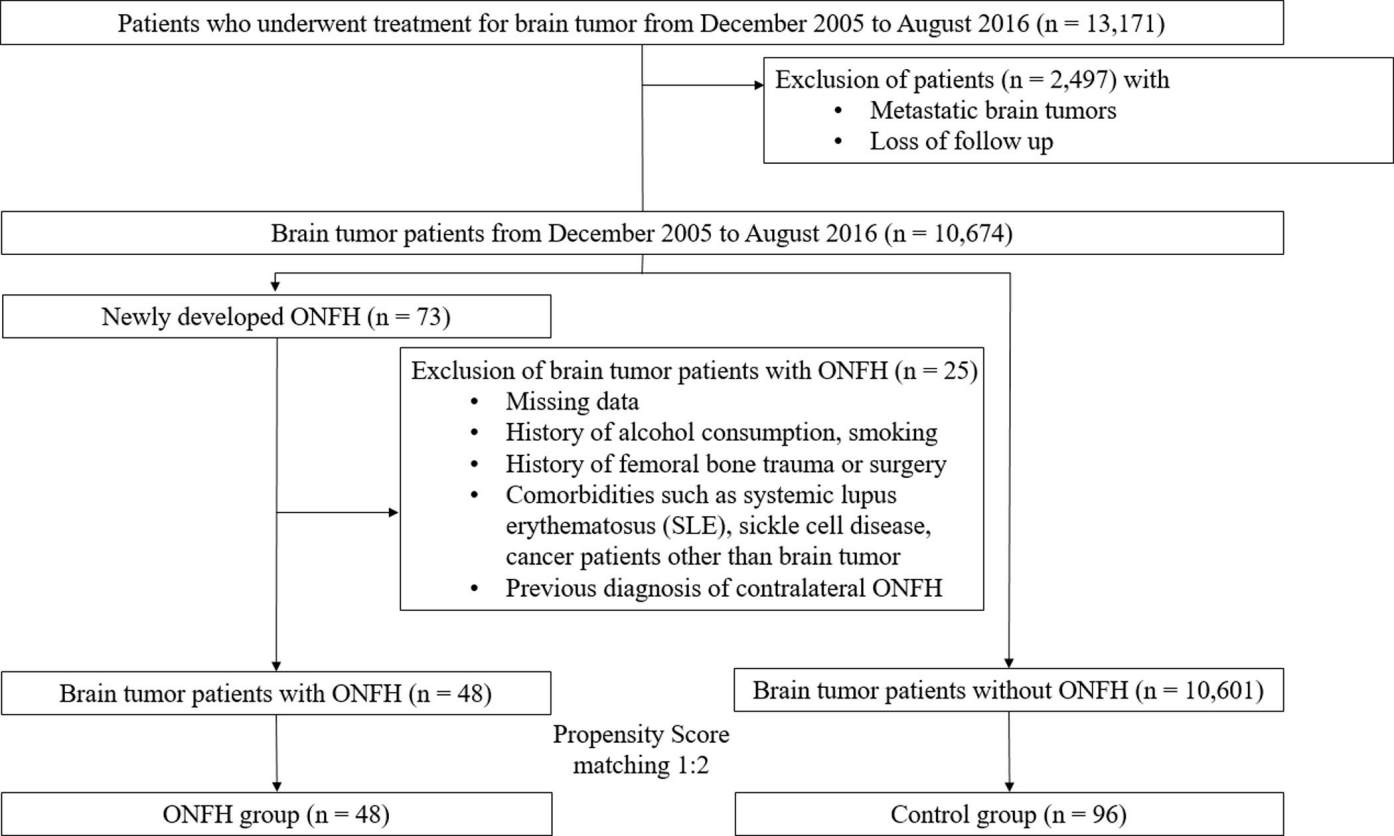
Flow chart of the study population. ONFH, osteonecrosis of the femoral head.

Our second objective of this study was to investigate risk factors of ONFH in primary brain tumor patients by comparing two groups using propensity score matched case-control study. Patients with newly developed ONFH were regarded as the ONFH group (case group), and patients without ONFH were regarded as the control group. To minimize factors other than those related to primary brain tumor itself and its treatment, we excluded cases (n = 25) with missing data, history of alcohol consumption, smoking, femoral bone trauma, or surgery, comorbidities such as systemic lupus erythematosus (SLE), sickle cell disease, cancer patients other than brain tumor, and previous diagnosis of contralateral ONFH from initial case group (n = 73) before entering the propensity score matching process (Fig 1). The baseline characteristics of the brain tumor patients with and without ONFH were obtained from the latest hospital electronic chart.

### Statistical analysis

Data are expressed as the mean and standard deviation for normally distributed continuous variables and as absolute numbers and percentages for categorical variables. Continuous variables were compared between the groups using the Student’s *t*-test or Wilcoxon rank-sum test. The chi-square or Fisher’s exact test was used to compare the distributions of categorical values between the groups. A 1:2 propensity score matching was performed to reduce potential bias between ONFH and control groups. A propensity score was generated for all patients using the logistic regression model. Variables used in this model included age, sex, body mass index (BMI), and follow-up period. We performed caliper matching on the propensity score (nearest available match). Pairs on the propensity score logit were matched within 0.2 standard deviation. Matching was performed by the minimal adjacent method of 1:2 pairing. Odds ratios (ORs) and corresponding 95% confidence intervals (CIs) were calculated using univariate and multivariate logistic regression analyses to determine risk factors of ONFH from primary brain tumor. The variables examined included the cumulative dose of corticosteroid (mg of corticosteroid as dexamethasone equivalents), comorbid conditions (diabetes and panhypopituitarism), location of the brain tumor (supratentorial or infratentorial), use of gamma knife surgery, brain radiotherapy, chemotherapy, and tumor removal surgery. Multivariate logistic regression analysis was performed by selecting variables that showed statistically significant difference through univariate logistic regression analysis. All statistical analyses were performed using SPSS Statistics, version 25.0 software (IBM Corp., Armonk, NY, USA). P-values <0.05 were considered significant.

### Ethical approval

This study was conducted under the approval of the institutional review board (IRB) of Samsung Medical Center (IRB Number: 2019-08-017). Because this study was a non-interventional retrospective study and all data were analyzed anonymously, the IRB waived the need for individual informed consent.

## Results

### Prevalence of ONFH in primary brain tumor patients

**Fig 2:**
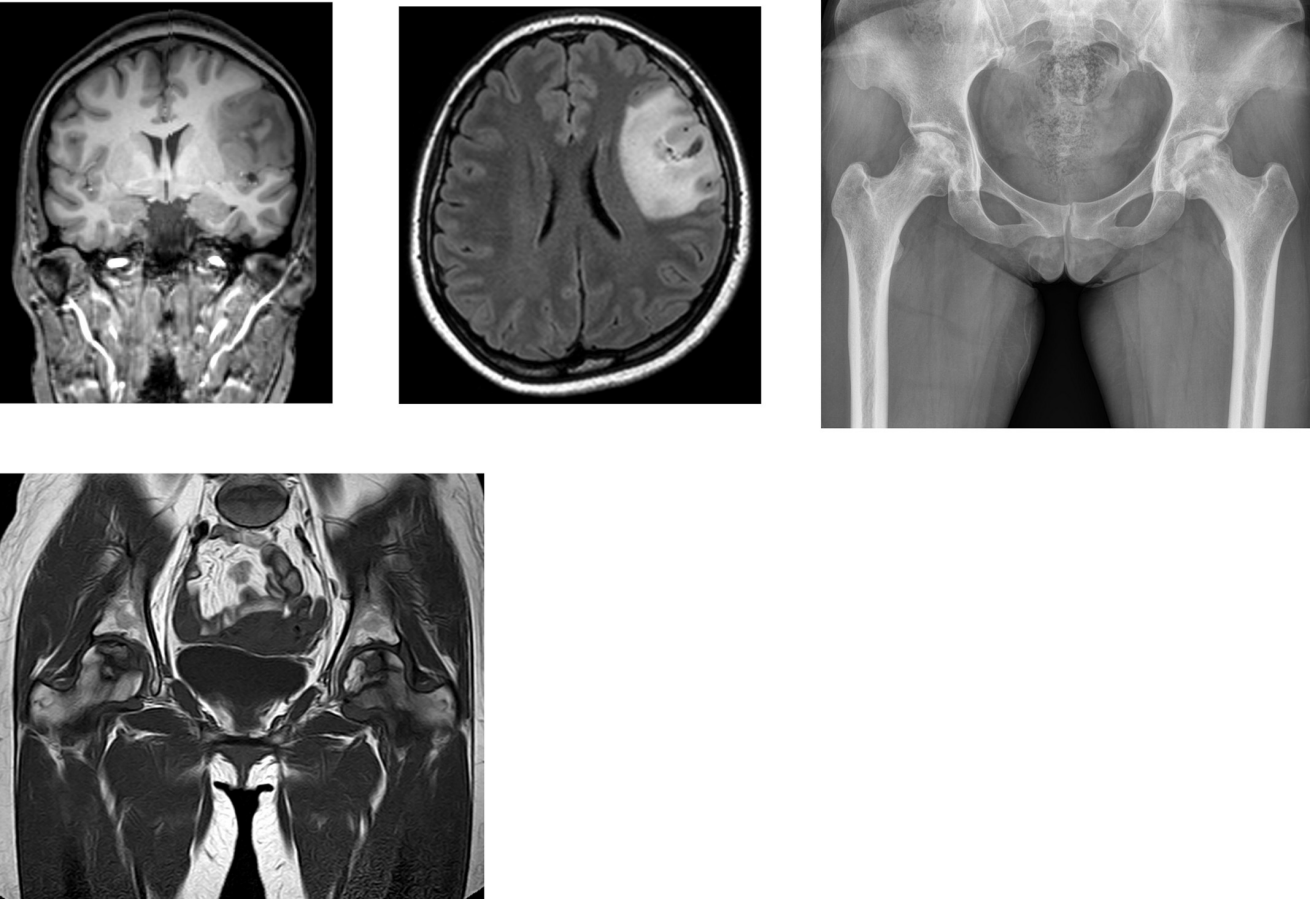
Example of a newly diagnosed osteonecrosis of the femoral head in brain tumor patient. (A, B) Brain magnetic resonance imaging (MRI) of a 25-year old woman who had generalized tonic–clonic seizures, revealed a tumor in the left frontal lobe. She was diagnosed with pathology-confirmed diffuse astrocytoma by navigation-guided biopsy. She received a total of 105 mg dexamethasone and underwent brain radiotherapy. (C, D) The patient suffered from hip pain 2 years after the astrocytoma diagnosis. Anteroposterior hip radiograph and MRI show osteonecrosis of the femoral head involving both hips.

The first objective of current study was to investigate the prevalence of ONFH in primary brain tumor patients. A total of 13,171 patients undergoing treatment for brain tumor between December 2005 and August 2016 were identified in our hospital. Patients who meet the initial exclusion criteria (n = 2,497) were excluded from this analysis; resulting in 10,674 patients with a primary brain tumor. Among them, we identified 73 patients (55 bilateral ONFHs in 73 patients) with newly developed ONFH during this study period (Fig 2), indicating that the prevalence of ONFH in primary brain tumor was 0.68%. The baseline characteristics of primary brain tumor patient with newly developed ONFH (n = 73) were as following: the mean age, BMI, and follow-up period after the brain tumor diagnosis were 42.11 (range, 16–59) years, 24.26 (range, 16–38) kg/m^2^, and 1,686.25 (range, 42–4,075) days, respectively. For the treatment of ONFH, 55 of 73 patients (75.3%) underwent THA on average 464.85 (range, 44–3522) days after the onset of hip pain.

### Risk factors related to ONFH in primary brain tumor patients

The second objective of this study was to determine risk factors of ONFH in primary brain tumor. The specific diagnoses of primary brain tumors are described in Table 1, and there was no statistically difference between two groups. Baseline characteristics such as age, sex, BMI, and follow-up period were similar between the two groups (P > 0.05). The baseline characteristics of the matched patient cohorts are summarized in Table 2.

**Table 1:** Comparison of brain tumors between the 48 brain tumor patients with ONFH (case) and 96 matched brain tumor patients without ONFH (control).

	Brain tumor patients with ONFH (n = 48)	Brain tumor patients without ONFH (n = 96)	*P*-value
Mengioma	10 (20.83%)	22 (22.92%)	0.821
Pituitary adenoma	7 (14.58%)	13 (13.54%)	0.882
Craniopharyngioma	6 (12.5%)	10 (10.42%)	0.738
Germinoma	5 (10.42%)	8 (8.33%)	0.708
Glioblastoma	4 (8.33%)	8 (8.33%)	1
Oligodendroglioma	3 (6.25%)	5 (5.21%)	1
Hemangioblastoma	3 (6.25%)	4 (4.17%)	0.689
Astrocytoma	3 (6.25%)	8 (8.33%)	1
Dermoid cyst	1 (2.08%)	1 (1.04%)	1
Acoustic neuroma	1 (2.08%)	0 (0%)	0.338
Pineal parenchymal tumor	1 (2.08%)	1 (1.04%)	1
Cavernous angioma 12	1 (2.08%)	3 (3.13%)	1
Langerhans cell histiocytosis	1 (2.08%)	1 (1.04%)	1
Vestibular schwannoma	1 (2.08%)	4 (4.17%)	1
Neurocytoma	1 (2.08%)	2 (2.08%)	1
Medulloblastoma	0 (0%)	3 (3.13%)	0.551
Ependymoma	0 (0%)	2 (2.08%)	1
Pituitary chondrosarcoma	0 (0%)	1 (1.04%)	1

ONFH, osteonecrosis of the femoral head.

**Table 2:** Demographic characteristics of the 48 brain tumor patients with ONFH (case) compared to 96 matched brain tumor patients without ONFH (control).

Baseline Characteristics	Brain tumor patients with ONFH (n = 48)	Brain tumor patients without ONFH (n = 96)	*P*-value
Patient age ^a^ (years)	36.44 ± 11.98	37.33 ± 18.91	0.730
Gender ^b^			1.000
Male	24 (50%)	48 (50%)	
Female	24 (50%)	48 (50%)	
BMI ^a^ (kg/m^2^)	24.29 ± 4.29	23.05 ± 4.38	0.109
Follow-up period ^a^ (days)	1859.83 ± 1351.31	1835.77 ± 1194.83	0.913
Cumulative dose of corticosteroid ^a^ (mg)	309.48 ± 258.78	335.43 ± 618.10	0.724
Number of brain tumor removal surgery ^a^	1.15 ± 0.55	1.03 ± 0.75	0.347
Gamma knife surgery ^b^	8 (16.67%)	34 (35.42%)	0.020
Brain radiotherapy ^b^	15 (31.25%)	20 (20.83%)	0.170
Chemotherapy ^b^	5 (10.42%)	24 (25.00%)	0.040
Supratentorial brain tumor ^b^	21 (43.75%)	22 (22.92%)	0.010
Comorbidities ^b^			
Diabetes	7 (14.58%)	2 (2.08%)	0.003
Panhypopituitarism	15 (31.25%)	9 (9.38%)	0.001
Tumor diagnosis to hip pain (days) ^a^	766.77 ± 617.18		
Tumor diagnosis to ONFH (days) ^a^	935.08 ± 632.42		

ONFH, osteonecrosis of the femoral head; BMI, body mass index.

a Values are mean and standard deviation.

b Values are the number of patients with percentages in parentheses.

For patients in the ONFH group, hip pain occurred after 766.77 (range, 43–2,331) days and ONFH was diagnosed after 935.08 (range, 86–2,556) days from the date of brain tumor diagnosis. In the ONFH group, the mean cumulative dose of corticosteroid (mg of corticosteroid as a dexamethasone equivalent) and the mean number of tumor removal surgeries were 309.49 mg (range, 0–1073 mg) and 1.15 (range, 0–2), respectively. Corticosteroids were administered within 24 hours after brain tumor surgery in 95.83% in the ONFH group and in 94.79% in the control group. In the ONFH group, the diagnosis of ONFH was made after 899.32 (range, 78–2,431) days from the date of brain tumor surgery. The proportions of the ONFH group with diabetes, panhypopituitarism, and supratentorial location of the tumor were 14.58%, 31.25%, and 43.75%, respectively. The proportions of the ONFH group who received gamma knife surgery, brain radiotherapy, and chemotherapy were 16.67%, 31.25%, and 10.42%, respectively. Specific chemotherapeutic agents in patients with chemotherapy after brain tumor surgery are listed in Table 3.

**Table 3:** Chemotherapeutic agents in brain tumor patients with adjuvant chemotherapy.

Chemotherapeutic agents	Brain tumor patients with ONFH (n = 5)	Brain tumor patients without ONFH (n = 24)
Temozolomide	2 (40%)	10 (41.67%)
Procarbazine, Lomustine, and Vincristine	1 (20%)	3 (12.5%)
Bleomycin, Etoposide, and Cisplatin	1 (20%)	2 (8.33%)
Etoposide, Ifosfamide, and Cisplatin	1 (20%)	1 (4.17%)
Cisplatin, Etoposide, Cyclophosphamide, and Vincristine	0 (0%)	2 (8.33%)
Methotrexate, Procarbazine, and Vincristine	0 (0%)	2 (8.33%)
Cytarabine	0 (0%)	2 (8.33%)
Others	0 (0%)	2 (8.33%)

ONFH, osteonecrosis of femoral head.

The values are given as the number of patients with the percentage in parentheses.

Regarding the risk factors of ONFH in primary brain tumor, there was a significant difference in univariate analysis, including in diabetes (OR = 8.024, 95% CI, 1.598–40.294, p = 0.011), panhypopituitarism (OR = 4.394, 95% CI, 1.794–11.008, p = 0.002), supratentorial location of brain tumor (OR = 2.616, 95% CI, 1.245–5.499, p = 0.011), and chemotherapy (OR = 2.867, 95% CI, 1.018–8.069, p = 0.046), while the cumulative dose of corticosteroid, the number of brain tumor surgeries, gamma knife surgery, and brain radiotherapy were not (Table 4). The multivariate logistic regression model revealed that diabetes (OR = 7.201, 95% CI: 1.349–38.453, p = 0.021) was an independent risk factors for ONFH in primary brain tumor patients (Table 5).

**Table 4:** Univariate logistic regression analysis of variables associated with the development of ONFH in brain tumor patients.

	Odds ratio (95% CI)	*P*-value
Cumulative dose of corticosteroid	1.000 (0.999–1.001)	0.780
Number of brain tumor removal surgery	1.274 (0.770–2.108)	0.345
Gamma knife surgery	0.365 (0.153–0.868)	0.230
Brain radiotherapy	1.727 (0.788–3.784)	0.172
Chemotherapy	2.867 (1.018–8.069)	0.046
Supratentorial brain tumor	2.616 (1.245–5.499)	0.011
Comorbidities		
Diabetes	8.024 (1.598–40.294)	0.011
Panhypopituitarism	4.394 (1.794–11.008)	0.002

ONFH, osteonecrosis of the femoral head; CI, confidence interval.

Data are odds ratios (95% confidence intervals).

**Table 5:** Multivariate logistic regression analysis of variables associated with the development of ONFH in brain tumor patients.

	Odds ratio (95% CI)	*P*-value
Chemotherapy	0.451 (0.151–1.344)	0.153
Supratentorial brain tumor	1.096 (0.362–3.319)	0.871
Comorbidities		
Diabetes	7.201 (1.349–38.453)	0.021
Panhypopituitarism	3.418 (0.923–12.654)	0.066

ONFH, osteonecrosis of the femoral head; CI, confidence interval.

Data are odds ratios (95% confidence interval).

Variables are adjusted for factors listed in the table.

## Discussion

ONFH is a devitalizing bone disease, characterized by collapse of the femoral head and subsequent loss of hip joint function [3, 4]. Numerous risk factors are associated with non-traumatic ONFH, including alcohol consumption and corticosteroid therapy [20, 21]. Additionally, fat cell hypertrophy, fat embolization, intravascular coagulation [1], and osteocyte apoptosis [22] have been suggested as important hypotheses for pathogenetic mechanism of ONFH. Several studies have investigated the association between ONFH and specific diseases to understand the pathogenesis of non-traumatic ONFH, in which bone cell death from compromised microvascular circulation is believed the result of trauma, corticosteroid, alcohol use, blood dyscrasias as well as other conditions such as Gaucher disease, Caisson disease, HIV, SLE, radiation treatment, pregnancy, inflammatory bowel disease, gout, smoking, and even malignant tumors [23–32]. Regarding the association with solid tumor, ONFH has been recognized as a complication of cancer chemotherapy for testicular, breast, ovarian, small-cell lung cancer and osteosarcoma, with prolonged and intensive steroid exposure usually implicated as the main risk factor [11–14, 33–35]. Despite chemotherapy and corticosteroid therapy frequently conducted for brain tumor treatment, insufficient studies have been performed to assess the association between ONFH and brain tumor. Only a few case reports and retrospective observational studies have been performed [16, 36–38], however, there is limited clinical information available regarding the relationship between ONFH and brain tumor. Although THA, a definitive surgical treatment for ONFH, can be a reliable one in brain tumor patients [19], ONFH occurring in brain tumor are still a grave concern for neurosurgeons as well as related professionals.

This study demonstrated a higher prevalence of ONFH in brain tumor patients (683.90 per 100,000) compared to those in previous studies conducted for general population with same ethnicity. Previous studies have reported ONFH prevalence per 100,000 persons among the general population as 28.91 in Korea (n = 14,103) and 8.92 in Japan (n = 11,400) [39–41]. Recent literature suggests that a sizable number of patients with a primary brain tumor suffer from corticosteroid-induced ONFH. Wong et al. [16] reported four cases of ONFH among 1,352 patients administered short-duration high-dose corticosteroid treatment. The risk of developing ONFH was 0.3%, with an incidence of 1 per 1,000 patient-years (4/total number of patients per year). Among the known risk factors of ONFH, the corticosteroid has been frequently used in brain tumor treatment for managing cerebral edema [21]. It is in this context that many neurosurgeons are concerned with corticosteroid-induced ONFH following in these patients. In terms of corticosteroid use, none of patients in this study meet the latest criteria of corticosteroid-induced ONFH [42], however, it has been recently emphasized that ONFH can occur even in those on low physiological corticosteroid replacement doses, most likely due to a combination of factors, including genetic susceptibility, corticosteroid dosage, and underlying condition [23, 25, 43]. In addition, oxidative stress, which has been suggested to play a role in the development of ONFH [44–46], might explain partly the association between ONFH and brain tumor. Brain is known to be particularly sensitive to oxidative injury due to the high content of polyunsaturated fatty acids, relatively low antioxidant capacity, low repair activity, the non-replicating nature of neuronal cells, a high rate of oxidative metabolic activity, and overproduction of reactive oxygen species (ROS) metabolites compared to other organs [47] In this study, the mean age of patients diagnosed with ONFH was relatively young, which is consistent with previous studies for the incidence of ONFH [48].

In our patients, hip pain appeared an average of 766.77 (range, 43–2,331) days after, and ONFH was diagnosed 935.08 (range, 86–2,556) days after, diagnosis of the brain tumor. Thus, a time lag occurred between the development of ONFH and its diagnosis. In younger patients, delaying progression with joint-preserving therapy (bisphosphonates, reduction of weight bearing) can reduce the need for multiple joint replacements. Indication for surgical versus non-surgical approach depends on the stage of disease, size of lesion, age and comorbidity of patients [49, 50]. Early detection and intervention are critical for joint-preserving therapy to increase chances of spontaneous resolution. Zhao et al. [51] reported that the median period from corticosteroid therapy to hip pain was 18 months in 269 patients with corticosteroid-induced ONFH and that 67% complained of hip symptoms within 24 months after commencing corticosteroid, which is comparable to our result. However, the time lag reported in this study was slightly longer than what has been reported for corticosteroid-associated ONFH by Koo et al. [52]. They reported that most ONFH occurred during the first 12 months after starting corticosteroid treatment with chemotherapy. The diagnosis of ONFH is often delayed because MRI is not always employed as part of the work-up when patients have pain. More importantly, a large number of ONFH patients remain asymptomatic during the early stage of ONFH. However, most patients who have large to moderate asymptomatic ONFH lesions frequently progress to requiring surgical treatment [53]. In this regard, the observed time from triggering event to hip pain and the diagnosis of ONFH in this study may be used as a practical reference for evaluating brain tumor patients.

In the present study, diabetes was associated with the occurrence of ONFH in primary brain tumor patients, with an OR of 7.201 in a multivariate logistic regression model. Although an insufficient number of studies have been conducted, Lai et al. [54] recently reported that Taiwanese patients with diabetes had a 1.16-fold increased risk for developing ONFH. Most risk factors for non-traumatic ONFH, such as steroid use, alcoholism, infection, marrow infiltrating diseases, coagulation defects, and some autoimmune diseases, are closely related to direct or indirect injuries to the bone vasculature [1, 55]. Microvascular and macrovascular insufficiency is a well-known complication caused by diabetes [56, 57], suggesting that vascular compromise of the femoral head related to diabetes may subsequently lead to ONFH in brain tumor patients [58]. Another potential hypothesis deals with oxidative stress associated with diabetes and brain tumors. Recent studies have implicated oxidative stress as a significant factor in the pathogenesis of vasculopathy and cancers and may have a role in the association between diabetes and ONFH in brain tumor patients. Several studies have supported a hypothesis regarding a role for *in vivo* oxidative stress in the pathogenesis of ONFH [7, 59, 60].

In contrast to previous studies, the cumulative dose of corticosteroid did not appear to affect the development of ONFH in brain tumor patients in the present study. Based on recently updated classification criteria for corticosteroid-induced ONFH, experts reached a consensus that patients should receive corticosteroid at a cumulative dose of ≥2 g over <3 months [42]. Considering that the corticosteroid doses as dexamethasone equivalents ranged from 0 to 1,073 mg (mean 309.49 mg) in the current study, ONFH that developed in this patient cohort would not meet the newly updated definition of corticosteroid-induced ONFH. However, regarding the role of corticosteroid in the pathogenesis of ONFH, contradictory results have been reported in the literature. Numerous studies have reported corticosteroid as a risk factor for ONFH [29, 61–63], and a few have shown that corticosteroid are not a risk factor for ONFH [64–66]. Although high-dose corticosteroid therapy was often associated with ONFH in patients with autoimmune diseases [40], mega-dose corticosteroid therapy did not induce ONFH in patients with a spinal cord injury [67]. Susceptibility to corticosteroid-induced ONFH may be dependent on multiple factors, including genetic background, the duration or amount of corticosteroid exposure, and underlying diseases [60].

The risk of the occurrence of corticosteroid-induced ONFH in brain tumor patients should not be underestimated. In univariate logistic regression, panhypopituitarism (OR = 4.394, 95% CI, 1.794–11.008, p = 0.002), supratentorial location of brain tumor (OR = 2.616, 95% CI, 1.245–5.499, p = 0.011) were associated with increased risk of ONFH. Our finding is supported by recent reports regarding the occurrence of ONFH in young patients following low-dose corticosteroid replacement therapy for panhypopituitarism secondary to surgery for brain tumors [17, 18]. The occurrence of ONFH with such a low dose of corticosteroid was surprising, however, Tokuhara et al. [68] observed that low levels of corticosteroid metabolising hepatic activity may increase responsiveness to corticosteroid and further risk of corticosteroid-induced osteonecrosis even with low corticosteroid dose in white rabbits.

In this study, chemotherapy was associated with the occurrence of ONFH in primary brain tumor patients (OR = 2.867, 95% CI, 1.018–8.069, p = 0.046). This finding is consistent with previous studies that have reported cytotoxic chemotherapy as a potential risk factor of ONFH in solid-tumor cancer patients [11–14]. Previously, meaningful amount of studies have reported the association between osteonecrosis and chemotherapy in testicular cancer, small cell cancer, breast cancer, ovarian cancer patients, which described the possible underlying pathophysiology and options available for its diagnosis, prevention and treatment [10, 11, 14]. The association between chemotherapeutic agents and osteonecrosis have been well demonstrated especially in pediatric cancer patients [69–71]. Moreover, several studies and case reports described osteonecrosis in a variety of cancers who underwent chemotherapy without receiving radiation or steroid therapy [69, 70, 72–75].

The results of the current study should be interpreted in light of several limitations. First, the retrospective study design risks introducing selection bias. In this study, sufficient data for matching was available in only 48 of the 73 brain tumor patients who were diagnosed with ONFH during the follow-up period among the 10,674 brain tumor patients, as our cohort was not a prospective one. However, we tried to reduce the effect of confounders by excluding cases with missing data, history of alcohol consumption or smoking, history of femoral bone trauma or surgery, comorbidities such as systemic lupus erythematosus (SLE), sickle cell disease, cancer patients other than brain tumor, and previous diagnosis of contralateral ONFH. Besides, propensity score matching was conducted to minimize selection bias. Second, the mean age of 36.44 years (1859.95 days) was relatively younger than the peak age of brain tumor onset, which might introduce the negative effect of improper propensity score matching. This can be explained by the fact that ONFH frequently occurs in young and middle-aged people, and also might be partly explained by the limitations of this sort of observational research. In this regard, identified ONFH in this study was confirmed through additional tests such as CT or MRI in the case of newly occurring symptoms such as hip pain or inguinal pain during the observation period of brain tumor patients. Considering the possibility that older patients tended to underreport their symptoms, younger mean age of this study could be interpreted as a possible factor of underestimation of the prevalence of ONFH in brain tumor patients. This might also emphasize the importance of exploring the association of brain tumor and ONFH, the main objective of this study. Lastly, owing to the heterogeneity in brain tumors and limited number of cases with ONFH, it was not possible to conclude whether the type and grade of brain tumor were associated with a greater risk of ONFH. In addition, it was difficult to clarify whether chemotherapy performed with corticosteroid therapy increased or decreased the risk of ONFH in this study. However, this study represents the largest homogenous primary brain tumor cohort with non-traumatic ONFH to date. Only a few studies are available that examine the association between ONFH and brain tumors. The results of the current study are the first to provide risk factors of ONFH in brain tumor patients as well as practical information regarding the prevalence and the time interval between brain tumor treatment and the development of ONFH, as a complication.

## Conclusions

This study demonstrated that the prevalence of ONFH in patients who underwent surgical resection of primary brain tumor was 0.68%. Diabetes was an independent risk factors for ONFH in these patients. Accompanying panhypopituitarism, supratentorial location of primary brain tumor, and chemotherapy were also associated in this study, whereas corticosteroid was not. In practice, prior to embarking on brain tumor treatment, counseling is required regarding the risk of developing ONFH and the orthopedic consequences. Vigilance is required by neurosurgeons and oncologists who must be aware of this complication.

## Supporting information

S1 Data (XLSX)
